# Barriers to Remote Health Interventions for Type 2 Diabetes: A Systematic Review and Proposed Classification Scheme

**DOI:** 10.2196/jmir.6382

**Published:** 2017-02-13

**Authors:** Michelle M Alvarado, Hye-Chung Kum, Karla Gonzalez Coronado, Margaret J Foster, Pearl Ortega, Mark A Lawley

**Affiliations:** ^1^ Department of Industrial and Systems Engineering Texas A&M University College Station, TX United States; ^2^ Department of Health Policy and Management School of Public Health Texas A&M Health Science Center College Station, TX United States; ^3^ Medical Sciences Library Texas A&M University College Station, TX United States

**Keywords:** diabetes mellitus, type 2, early medical intervention, biomedical technology, remote sensing technology, terminology as topic

## Abstract

**Background:**

Diabetes self-management involves adherence to healthy daily habits typically involving blood glucose monitoring, medication, exercise, and diet. To support self-management, some providers have begun testing remote interventions for monitoring and assisting patients between clinic visits. Although some studies have shown success, there are barriers to widespread adoption.

**Objective:**

The objective of our study was to identify and classify barriers to adoption of remote health for management of type 2 diabetes.

**Methods:**

The following 6 electronic databases were searched for articles published from 2010 to 2015: MEDLINE (Ovid), Embase (Ovid), CINAHL, Cochrane Central, Northern Light Life Sciences Conference Abstracts, and Scopus (Elsevier). The search identified studies involving remote technologies for type 2 diabetes self-management. Reviewers worked in teams of 2 to review and extract data from identified papers. Information collected included study characteristics, outcomes, dropout rates, technologies used, and barriers identified.

**Results:**

A total of 53 publications on 41 studies met the specified criteria. Lack of data accuracy due to input bias (32%, 13/41), limitations on scalability (24%, 10/41), and technology illiteracy (24%, 10/41) were the most commonly cited barriers. Technology illiteracy was most prominent in low-income populations, whereas limitations on scalability were more prominent in mid-income populations. Barriers identified were applied to a conceptual model of successful remote health, which includes patient engagement, patient technology accessibility, quality of care, system technology cost, and provider productivity. In total, 40.5% (60/148) of identified barrier instances impeded patient engagement, which is manifest in the large dropout rates cited (up to 57%).

**Conclusions:**

The barriers identified represent major challenges in the design of remote health interventions for diabetes. Breakthrough technologies and systems are needed to alleviate the barriers identified so far, particularly those associated with patient engagement. Monitoring devices that provide objective and reliable data streams on medication, exercise, diet, and glucose monitoring will be essential for widespread effectiveness. Additional work is needed to understand root causes of high dropout rates, and new interventions are needed to identify and assist those at the greatest risk of dropout. Finally, future studies must quantify costs and benefits to determine financial sustainability.

## Introduction

### Background

Management of type 2 diabetes requires healthy lifestyle habits including diet, medication adherence, and exercise. Thus, patients must practice strong *self-management*, the act of taking responsibility for one’s own behavior and well-being. Conventional outpatient therapies fail to address the daily decision-making challenges faced by patients with diabetes [1]. Thus, providers have begun experimenting with remote health to help patients further manage their conditions. *Remote health* is a type of ambulatory health care that allows patients to use technology to collect data and communicate with their health care provider in a different location. We use the term *remote health intervention* to refer to the specific remote health actions and technology employed to improve patient health. This paper focuses on remote health interventions for type 2 diabetes self-management. These remote health interventions can provide a window into the patient’s daily activity levels, medication adherence, diet habits, and health vitals. In theory, this transparency enables proactive intervention for poor compliance and emerging risks, assuring better daily health and helping patients avoid hospital visits. This paper assumes that remote health encompasses any type of health care delivered remotely, including telemonitoring, telemedicine, telehealth, eHealth, and mHealth.

Research shows several benefits including high levels of patient satisfaction, positive behavioral changes, and better health outcomes (both physical and mental) [2-4]. Unfortunately, researchers have discovered many barriers to implementation that must be resolved before payers invest in full-scale adoption [5]. This paper systematically reviews the research literature to identify observed barriers to remote health implementation, adoption, and retention for adult patients with type 2 diabetes in the United States. This review was restricted to a single country because of the substantial differences at the financial, strategic, operational, and tactical levels of health care systems and settings in different countries. Payment structures and technology access are 2 such conditions that frequently vary between countries. The barriers identified in this systematic review will inform the design and implementation of future remote health interventions for diabetes self-management in the United States. Researchers in other countries will also be more informed about barriers to remote health for diabetes from the results of this review, although the prevalence of each barrier category will undoubtedly vary from one country to another.

### Literature Review

This review identified 24 other relevant reviews. Although these reviews provided no systematic analysis of barriers, it is important to compare and contrast their contributions with ours.

Reviews focused on diabetes outcomes for the general population reported improved health for patients using telemedicine as compared with those with regular care [6-10], whereas those focused on outcomes for specific populations saw mixed results [11-13]. Baig et al [11] addressed African American and Hispanic diabetic patients and concluded that rigorous evaluation of existing and new interventions was badly needed. Van den Berg et al [12] found that most elderly patients living at home were able to use telemedicine devices to good effects, whereas Sutcliffe et al [13] found no conclusive evidence that communication technologies improved health outcomes for younger patients.

Other reviews considered specific types of technology such as mobile phones and apps [14-16]. Baron et al [14] found methodological weaknesses and inconsistent evidence, whereas Holtz and Lauckner [15] saw positive trends resulting from mobile phone use. Chomutare et al [16] identified the most prevalent features of mobile phone diabetes apps and found personal education to be the most underrepresented. Arnhold et al [17] evaluated mobile phone app usability and found apps with analysis functions to have the least favorable usability scores. Three reviews focused on telehealth for the broader area of chronic disease management. Dennis et al [18] found that telephone coaching improved health behaviors, self-efficacy, and health status, especially for vulnerable populations with limited access to health services. Hamine et al [19] found mixed evidence and called for more research on overcoming barriers, and van den Berg et al [12] found insufficient customization in telemedicine apps for older adult populations, a barrier identified in our study as well. Two reviews investigated how information technology affects diabetes self-management. El-Gayar et al [20] reported the need for more comprehensive, user-centered interventions, whereas Cotter et al [21] found Internet interventions to be viable options for diabetes self-management, especially those with personalized feedback, tracking, and peer support.

Two reviews analyzed behavioral telehealth interventions for glycemic control. Cassimatis and Kavanagh [4] found that behavioral telehealth interventions show promise in improving diabetes self-care and glycemic control, especially those emphasizing physical activity and dietary adherence. Behavioral change techniques such as feedback on performance, education on consequences, and self-monitoring were linked to positive changes in health behaviors, psychological well-being, and clinical parameters [3]. Two other reviews explored the cost of telemedicine for diabetes self-management and found little evidence to support claims of cost-effectiveness [1,22]. Greenwood et al [23] concluded that telehealth interventions rarely include all the elements of the care protocols recommended by the International Diabetes Federation. Lepard et al [24] compared telehealth with face-to-face interventions for rural adults with type 2 diabetes and found collaborative goal-setting to be effective for both.

Wilson et al [25] examined the barriers and facilitators of access to self-monitoring for minority populations. Cultural awareness, social expectations, and language were identified as barriers. Our study did not reveal these same barriers to be as prevalent, presumably due to reporting bias from the provider’s perspective as opposed to the patient’s perspective. In addition, each study design only included patients who could fluently speak the language used by the remote health intervention. Finally, a review by Radhakrishnan et al [26] identified barriers and facilitators for sustainability of telehomecare programs for chronic disease management, including barriers regarding health literacy of the patients and cost-effectiveness of remote health. Some of these same barriers were identified in our search as well.

In this research, we develop an inventory of common barriers to the implementation of remote health interventions over the last 5 years. In addition, we analyze the frequency of each barrier type, outcome measures, terminology, and technology used. We also discuss dropout rates, payments to patients, reported costs of the remote health interventions, and suggestions to overcome barriers. Finally, we draw connections between our barrier inventory and a newly proposed conceptual model of necessary conditions for successful remote health.

### Objective

The objective of this systematic review was to identify and classify barriers to remote health interventions for adult patients with type 2 diabetes in the United States. We define a *barrier* as any cause of reduced technology effectiveness.

## Methods

This section describes retrieval procedures, inclusion and exclusion criteria, and the data collection process of this systematic review.

### Retrieval Procedures

We searched the following 6 electronic databases: MEDLINE (Ovid), Embase (Ovid), CINAHL, Cochrane Central, Northern Light Life Sciences Conference Abstracts, and Scopus (Elsevier). The focus was on 2 primary concepts that were searched using keywords and appropriate index terms for each database: type 2 diabetes (diabetes mellitus, type 2, type II) and remote health (telemonitor, telemedicine, telehealth, mHealth, eHealth). Several variations of the term “noninsulin dependent” were included because this is another way to describe type 2 diabetes. Multimedia Appendix 1 provides specific queries for Medline, Embase, and CINAHL databases. Results were limited to papers published in English from 2010 to 2015. Articles were exported into Refworks (ProQuest) and duplicates removed. We also used Scopus (Elsevier) to search references and citations of included studies.

### Inclusion and Exclusion Criteria

Each article was screened by 2 independent authors in Refworks, first by the title and abstract, then by the full text. To be included, a study had to (1) involve patients with type 2 diabetes; (2) show results of an implemented study or trial; (3) use remote health (or the terms mHealth, eHealth, telemonitor, telehealth, or telemedicine); (4) occur in the United States; and (5) use adult populations. We excluded all duplicate texts and articles without available full texts. In addition, articles that were editorials, systematic reviews, article reviews, personal opinion articles, case studies, and all other summary-type or synthesis-type articles were excluded [27]. Any discrepancies between the 2 reviewers were settled by a third reviewer or group consensus.

### Data Collection Process and Data

Reviewers worked in teams of 2 to code data from 53 papers using Google Forms. The form was created by 3 authors and then tested and revised by all authors prior to use. After the finalization of the Google Form, the 2 reviewers worked together to code each paper, discussing and agreeing on all the information before recording. Information was collected on study characteristics (time, length, location, type, facility, gender, participant age, socioeconomic status, eligibility, number of participants, costs, languages used), outcome measures, dropout rates, key results, remote health intervention name, technology used, barriers to implementation, and suggestions to reduce or eliminate the barriers.

## Results

### Results of the Study Selection

**Figure 1:**
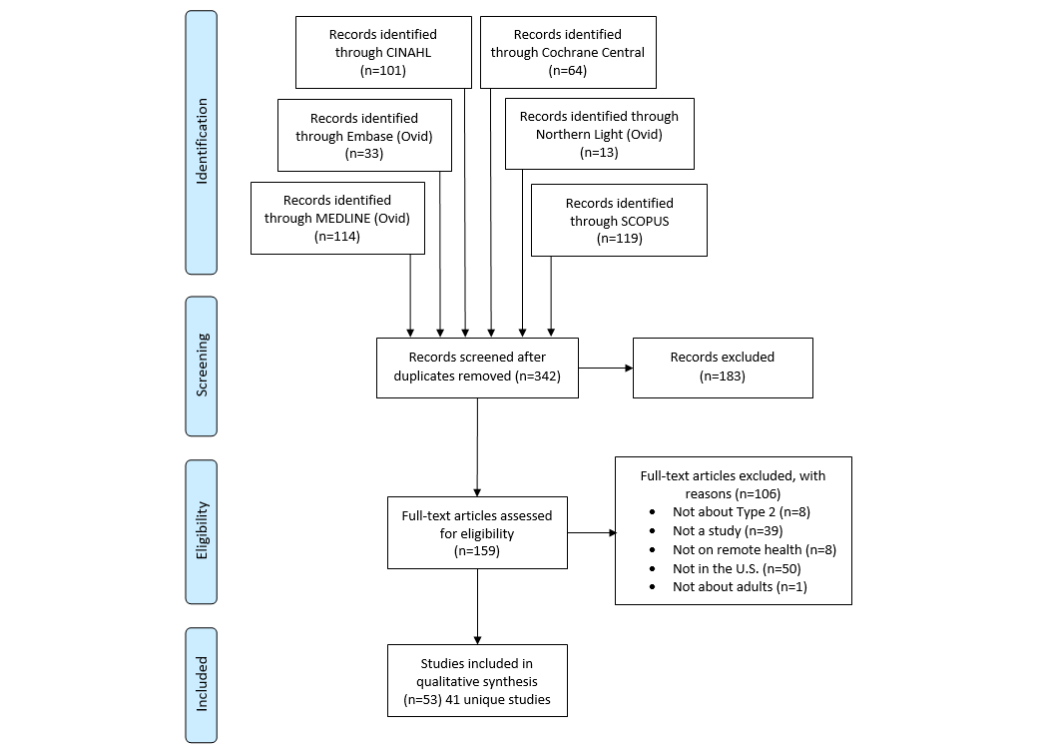
Selection process.

Figure 1 shows the study selection process. The initial search resulted in 342 citations from 6 databases. The original searches yielded 114 articles from Medline, 33 from Embase, 101 from CINAHL, 64 from Cochrane Central, 13 from Northern Light, and 119 from Scopus. There were 159 articles that passed title and abstract screening. From these, 106 articles were eliminated during full-text screening. The reasons for exclusion of the articles were as follows: 8 were not about type 2 diabetes, 39 were not studies, 8 were not about remote health, 50 were not in the United States, and 1 was not about adults. There were 53 articles that implemented remote health interventions for self-management of adult type 2 diabetes in the United States published from 2010 to 2015. Some of the publications were on the same project (IDEATel, TExT-MED, and Beacon Communities), so information for those articles was combined for a total of 41 unique studies that met search criteria. See Multimedia Appendix 2 for a summary of these articles and reports on findings for a limited number of categories (eg, outcome measures, technology used, barriers).

### Study Characteristics

This section describes the characteristics of studies included in our systematic review. The following subsections analyze the types of studies, length of studies, study dates, participant characteristics (age, gender), study locations, languages, comorbidities, types of technology, terminology, outcome measures, and costs.

#### Types of Studies

Of the included studies, 51% (21/41) were randomized controlled trials (RCT), 24% (10/41) were pilot studies of a newly developed remote health intervention, and 10% (4/41) were quasi-experimental studies (Multimedia Appendix 3). One study [28] identified itself as a quasi-experimental pilot study, and thus was classified as both in our analysis. The “Other” category had the following entries: longitudinal study, nonrandomized parallel control group study, observational study, cluster-randomized clinical trial, prospective randomized trial, nonrandomized prospective observational preintervention or postintervention studies, and prospective longitudinal randomized trial.

#### Length of Study

Study length ranged from 2 weeks [29] to 5 years [30]. As shown in the Multimedia Appendix 3, % (21/41) of the papers were shorter than 6 months and 34% (14/41) of the studies lasted between 7 and 12 months. Only 12% (5/41) studies lasted more than a 1-year period. One study did not report its length [31,32]. The longest studies were 3.5 years [33] and 5 years [30].

#### Study Dates

All articles were published from 2010 to 2015, as required by the search criteria. There were 83% (34/41) studies that provided specific dates in which the study occurred. Of the studies that reported this information, all occurred from 2000 to 2013, with the earliest occurring during 2000 to 2005 [30].

#### Participant Characteristics

##### Number of Participants

The number of participants in the study varied from 11 [34] to 1838 [35,36] (Multimedia Appendix 3). The majority of studies (71%, 29/41) had less than 200 participants, and 10% (4/41) had more than 400 participants [30,35-38]. One study did not report the number of participants [39].

##### Participant Age

All studies were conducted on patients above 18 years of age (as specified in the inclusion criteria). One study reported the mean participant age in the 30s [40], 12% (5/41) studies in the 40s, 46% (19/41) in the 50s, and 20% (8/41) in the 60s (Multimedia Appendix 3). A single study had participants with mean age in the 70s [30]. There were 17% (7/41) studies [33,38,39,41-44] that did not report the mean participant age. Three studies [33,41,42] only provided participant age range.

##### Gender

Most studies (76%, 32/41) had a female to male ratio ranging from 38:62 [45] to 81:19 [33]. Stone et al [44] had only male participants, whereas 2 other studies [34,40] were mostly female. There were 12% (5/41) Veterans Affairs (VA) studies that had less than 7% female participants. There were 7% (3/41) of the selected studies [39,42,43] that did not report gender.

#### Location

The selected studies were analyzed by the geographic location in the United States using regions defined by the US Census Bureau [46]. After analyzing the selected studies by geographic regions, more studies (37%, 15/41) were conducted in the Midwest than any other region.

Multimedia Appendix 3 indicates that 22% (11/41) studies were conducted in the west, 32% (13/41) studies were conducted in the south, and 22% (9/41) studies were conducted in the northeast region. Note that the numbers in Multimedia Appendix 3 sum to 48 because some studies were in multiple states, for example, study [39] had locations in 3 states, viz, UT, LA, and MI.

#### Language

Most of the communications in the studies were exclusively English-based (71%, 29/41). A single study had a primary language other than English and was conducted in Spanish [33]. However, a considerable portion of the studies (22%, 9/41) were implemented with both English and Spanish options available for patients. Two studies [47,48] were available in English, Spanish, and Cantonese. No other languages were incorporated as part of the studies observed.

#### Comorbidity

The majority of papers (74%, 31/41) focused solely on type 2 diabetes. Others studied diabetes patients with comorbidities, including 12% (5/41) with hypertension and 7% (3/41) with cardiovascular conditions. Patients in the study by Henderson et al [42] exhibited comorbidities in hypertension, hyperlipidemia, and cardiovascular disease; patients in Dang et al [28] had comorbid hypertension and osteoarthritis; and patients in Abebe et al [30] had comorbid cognitive decline. One study included both type 1 and type 2 diabetes [49]. Multimedia Appendix 3 summarizes these findings.

#### Technology

Studies used between 1-8 different types of technology (median=3) for remote health interventions. The following 9 technology categories were identified: Phone-Voice, Phone-Text, Mobile Device-Internet or Apps, Video, Email (computer or mobile phone), Remote Health Unit, Computer or Internet, Glucose Monitor, and “Other Health Device.” Phone-Voice indicates that phone calls were made via landlines or mobile phones. Phone-Text refers to the use of text messages as a communication method between the researchers and the participants. Mobile Device-Internet or Apps indicates the study allowed the participant to access health data through Internet or an app via mobile phones, iPads, or tablets.

Video involved the use of videoconferencing, but could be done through a mobile phone or tablet app or a laptop’s integrated camera, a desktop computer with a camera, or a remote health intervention with videoconferencing capabilities. Email was created as a category because many studies mentioned the use of email as a communication form but did not specify how it was to be accessed (eg, mobile device, laptop, desktop). Remote health units were single devices, often developed commercially, that combined different types of technology for both monitoring and communication. The Computer or Internet category indicates data were sent over the Internet using a computer, laptop, or other device. “Other Health Device” indicates the use of a health monitoring device, other than a blood glucose monitor, such as a blood pressure monitor or telemetry device. Glucose monitors were counted as a unique category as they are highly associated with diabetes self-management.

**Figure 2:**
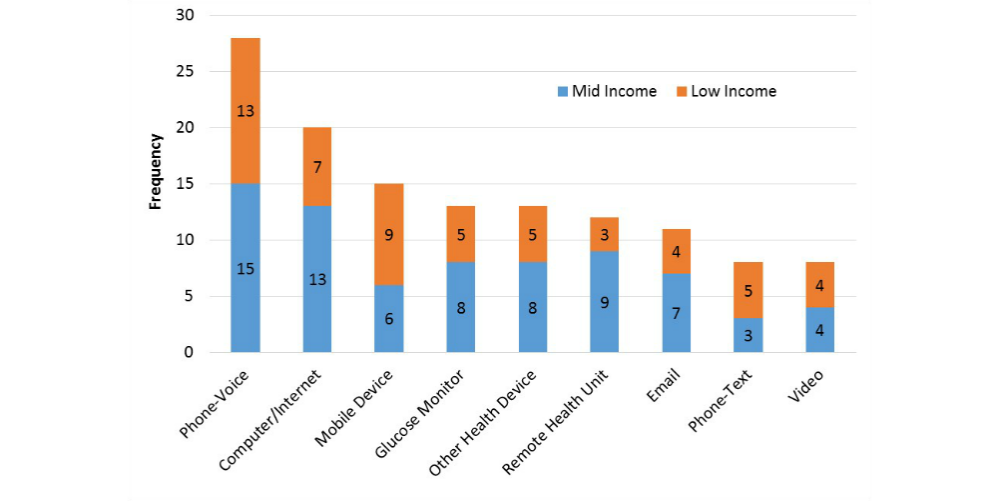
Technology used by popularity.

Figure 2 shows the utilization of different types of technology used in the low- and mid-income studies. Phone-Voice was used by 68% (28/41) of the studies and Computer or Internet was used by 49% (20/41) of the studies. Also, 37% (15/41) of the studies mentioned using a mobile device for Internet or app access. There were 29% (12/41) studies that used a remote health unit like the Authentidate Electronic House Call [50]. Only 5 low-income studies used a blood glucose monitor compared with 8 regular income studies. Finally, 20% (8/41) studies used video conferencing in their program and 27% (11/41) used email as a source of communication.

#### Terminology

**Figure 3:**
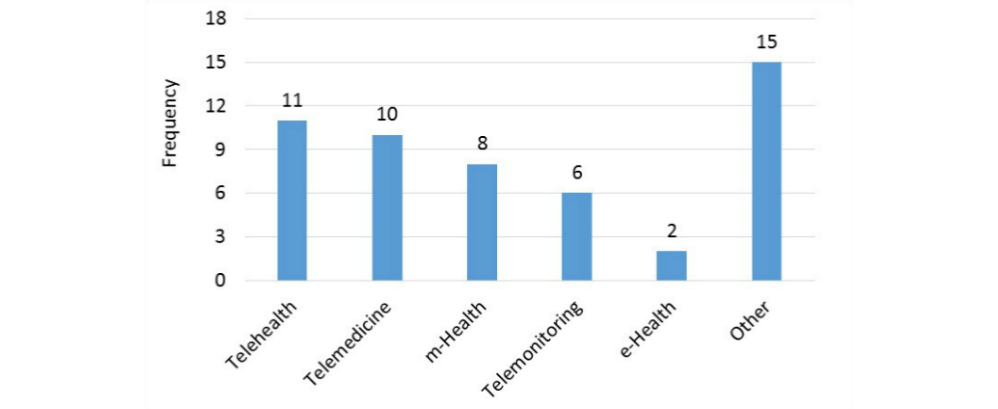
Terminology popularity index.

There was a variety of terminology used to describe the remote health intervention used in each study (Figure 3). There were a total of 20 unique terms. The most popular term found in our articles was “telehealth” which was used in 27% (11/41) of the studies. This was followed by “telemedicine” which was mentioned in 24% (10/41) of the articles and “m-Health or mHealth” was the primary term for 20% (8/41) of the studies. However, there were 15 terms in the “other” category because each was only found in a single paper. These “other” terms were: telecommunications, televisits, text-messaging intervention, voice intervention, automated telephone, Web-based, medical assisted coaching (MAC), automated telephone self-management support program, home health monitoring, text-message based program, mobile phone personalized behavioral intervention, teleconsultation, diabetes care telemonitoring device trial, remote monitoring, and telephone care management.

#### Outcome Measures

**Figure 4:**
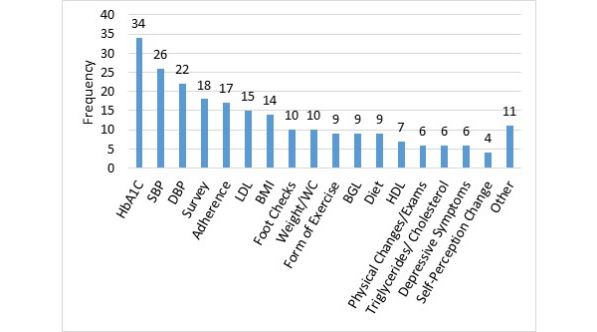
Outcome measures.

Each study used between 1 and 16 outcomes measures (median=5) to evaluate the effectiveness of the remote health intervention. The outcome measures varied across studies. The commonly used outcome measures are tallied in Figure 4. HbA1C was the most prevalent outcome measure and 83% (34/41) of the papers utilized this measure. Davis et al [51] referred to glycated hemoglobin as GHb and used it to evaluate the effectiveness of telehealth diabetes self-management, but we considered this as the same term as HbA1C. Blood glucose level was an outcome measure for 22% (9/41) of the papers. All the papers that measured the sugar level also included HbA1C counts except Aikens et al [52].

There were 63% (26/41) of the studies that measured blood pressure. The 11 of 12 low-income studies measured systolic blood pressure (SBP) and diastolic blood pressure (DBP). But of the 14 mid-income papers that checked SBP, only 11 checked DBP [34,53,54]. High-density lipoprotein (HDL) was not as frequently seen as an outcome measure as low-density lipoprotein (LDL). HDL is popularly known as the “good cholesterol” and physicians are more concerned with regulating the high-level of LDL or “bad cholesterol” in patients’ blood. For example, of the 37% (15/41) papers that recorded LDL, only 7 also measured HDL outcomes. Body mass index (BMI) was reported as an outcome measure in 34% (14/41) of the papers. Figure 4 combines waist circumference and weight into a single category which was used in 24% (10/41) of the studies.

Self-efficacy or adherence either in diet, exercise, medication, or diabetes management was an outcome measure in 41% (17/41) of the studies. This was labeled in Figure 4 as “Adherence.” For instance, there was self-efficacy in Diabetes Management Practices Scale [55]; adherence in immunizations [40]; the Medication Adherence Self-Efficacy Scale [29,56]; and adherence to the use of home telemedicine unit, home monitoring, and diabetes health maintenance [30]. Physical Changes or Exams (labeled “Physical Exams” in Figure 4) were performed in 15% (6/41) of the papers. The type of physical exam varied greatly from the Framingham Risk Score [28], Albumin-to-creatinine ratio [51], glycemic control [57], and Charlson Comorbidity Index [30]. None of the mid-income studies had Physical Changes or Exams as outcome measures as opposed to 6 low-income studies. Furthermore, 24% (10/41) of the studies included foot checks, and 22% (9/41) checked for diet and exercise levels.

There were 43% (18/41) of the studies that used surveys, interviews, and mixed methods to gather information on participants. Some studies [29,39,57] implemented surveys to understand the patient’s satisfaction rate with the programs. Others sought to gage the participant’s knowledge level on diabetes medication [29]. Ruggiero et al [58] implemented a survey to understand the environmental barriers participants experienced that prevented patients from taking their medication. Finally, it should be noted that there were less frequently used “other” outcome measures noted in 27% (11/41) of the studies that gathered information on weekly activities, Self-monitoring of blood glucose (SMBG) measurement data, fat intake, fructosamine blood level, dilated eye exam, diabetes distress, microalbumin, health literacy, trigger safety concerns, alcohol intake, and smoking status, such as Palmas et al [30] and Pressman et al [45].

#### Cost

In the review, 88% (36/41) of the studies did not mention cost estimates of the remote health intervention. Davis et al [51] and Henderson et al [42] described a method to compute costs but did not report results. Only Palmas et al [30] documented costs, estimating a total of US $622 per person/month. This cost was US $358/month from technology vendors, US $115/month from the bioinformatics team, and US $149/month from the clinical teams. However, this estimate did not account for participant’s transportation savings due to the remote health intervention. Fischer et al [38] reported an overall direct programmatic cost of US $134,750 for the 20-month intervention. Greenwood et al [23] reported US $34.20 per participant for phone and messaging over 9 months, and Katalenich et al [59] varied depending on the intervention; however, these ranged from US $12.52 to US $15.50 per person.

### Main Results

In this section, we define 5 categories of barriers, catalogue and inventory of each barrier, discuss barrier impacts, and present a conceptual model of conditions under which remote health can be successful and sustainable.

#### Barrier Classification

After identifying barriers to successful remote health interventions among the included studies, the authors then organized the barriers into 5 categories based on their relation to a resource (*patient, provider, health system, digital infrastructure, or intervention design*). *Patient (P)* barriers are the causes of reduced effectiveness originating with the patient, such as health illiteracy. *Technology Access (T)* barriers arise from restricted availability of supporting digital infrastructure, such as poor wireless coverage. *Design (D)* barriers are shortcomings in the specified technical configuration and intended mode of use, such as inadequate support for provider feedback. *Provider (Pv)* barriers are those originating with the provider’s care team and environment, such as staff training, and *System (S)* barriers include health care organization factors external to the care environment that affect technology effectiveness, such as limited institutional support. All identified barriers fell within these categories. Furthermore, barrier prevalence was related to income level and thus this information was recorded. Table 1 provides an inventory of barriers identified, organized by these types, along with income level.

**Table 1:** Barrier inventory.

Barrier type			Income level		
			Low	Mid	Total
**Patient barriers**					33
	P1	Low formal education	4	0	4
	P2	Technology illiteracy (uncomfortable with technology)	7	3	10
	P3	Medication nonadherence	3	2	5
	P4	Patients desire in-person contact with provider (perceived lack of confidence and comfort)	3	0	3
	P5	Low perceived value or effectiveness	2	2	4
	P6	Health illiteracy	4	1	5
	P7	Other	1	1	2
**Technology access barriers**					21
	T1	Patient does not have required technology	5	3	8
	T2	Technology is cost prohibitive to the patient (not affordable)	4	1	5
	T3	Limited internet access in the area	3	0	3
	T4	Other	3	2	5
**Design barriers**					60
	D1	Lack of customization to patient preferences and needs	5	3	8
	D2	Lack of accuracy or reliability (patient or provider)	7	6	13
	D3	Content not engaging or relevant	3	6	9
	D4	Timing of patient-provider interactions	2	1	3
	D5	Decisions of content and frequency of interventions	3	3	6
	D6	Patients not incorporated into the design needs	3	0	3
	D7	No analysis on impact with comorbidities	2	1	3
	D8	Labor- and time-intensive for providers	4	2	6
	D9	Other	4	5	9
**Provider barriers**					14
	Pv1	Data accessibility to patient logs (access to patient logs)	2	1	3
	Pv2	Low integration into provider work flow	3	1	4
	Pv3	Other	3	4	7
**System barriers**					20
	S1	Limitations on scalability	1	9	10
	S2	Lack of program reimbursement by insurance	1	2	3
	S3	High cost of intervention	1	2	3
	S4	Other	3	1	4

#### Barrier Inventory

Each of the 41 included studies reported or showed evidence of 0-12 barriers (median=3, mean=3.6). Three studies [33,45,60] discussed limitations of the study designs, but did not report barriers associated with design and implementation of the remote health intervention.

##### Patient Barriers

*Patient Barriers* (P1-P7) were identified in 33 studies. Technology illiteracy (uncomfortable with technology) was reported in 24% (10/41) of the studies, whereas low health literacy and low formal education were reported in 12% (5/41) and 10% (4/41) of the studies, respectively. Several authors noted that VA populations tended to be more technology savvy than general populations [52,54,61-63]. In many instances, patients could use computers and the Internet, but felt uncomfortable or lacked confidence in Internet-based communication and preferred using phone calls.

##### Technology Access Barriers

*Technology Access Barriers* (T1-T4) were identified 21 times. The main barrier (20%, 8/41) was that the patient did not own or have access to the required technology. For instance, Arora et al [49] eliminated 51 of 74 potential candidates because they did not have a text-capable mobile phone. In some cases, the patient could not afford to purchase the technology (12%, 5/41). Consequently, studies either limited participation to patients able to afford the technology or it was provided. It was noted that some patients started with the technology but eventually dropped out due to costs. In other instances, the patient had the technology available but had limited access to the Internet (7%, 3/41).

##### Design Barriers

*Design Barriers* (D1-D9) were noted 61 times. The most common *Design Barrier* was inaccurate or unreliable data (32%, 13/41), as most studies required patients to manually enter their own data and were therefore prone to recall bias or human error. A total of 22% (9/41) studies reported issues with the content of the intervention not being engaging or relevant, and 20% (8/41) reported a lack of customization of the intervention. This was particularly true for studies that utilized manual input or did not provide language alternatives for nonnative English speakers.

##### Provider Barriers

*Provider Barriers* (Pv1-Pv3) were referenced in 14 studies, the most common being poor integration of remote health technology and provider work flow (10%, 4/41). There were physician complaints of the system being intrusive [53,64] and a single study described the work flow challenges arising from the call center not being located near the clinical areas [41]. Personnel shortage and insufficient training were also mentioned, as was limited transparency of patient health data [53,64]. The latter most commonly occurred in systems relying on manual data collection and uploads where the patient failed to provide the data at the time of communication with the providers.

##### System Barriers

*System Barriers* (S1-S4) were reported 21 times with the most common being limitations on scalability in 24% (10/41) studies. Scalability issues were most commonly cited because the study involved only a few providers or had specialized populations (eg VA patients), and the authors were unsure of how the results would apply to larger, more general populations. There were 6 S *ystem Barriers* classified as “Other” because they were less common and included high cost of interventions [30], undiversified population of VA patients [28], unreported cost-effectiveness [57], and an uncontrolled study design [49].

Most barriers occurred in both low- and mid-income populations, but some were more prevalent in one population than the other. For example, *Patient Barriers* were more prevalent in low-income studies which had 24 instances compared with the mid-income studies that had only 9 instances, with low-income patients having more difficulty using the technology. *System Barriers* were most prominent in mid-income studies (14 times) as compared with low-income (6 times) with limitations on scalability to larger population sizes being the most prevalent barrier.

#### Barrier Impacts

##### Dropout Rates

The patient dropout rate was a commonly reported problem ranging from 5% to 57% and averaging 22%. Buis et al [35,36] experienced the largest at 57%. There were 27% (11/41) studies that did not report a dropout rate [23,28,34,38,39,42,44,59,62,65,66]. Multimedia Appendix 3 provides a histogram of dropout rates. There were 14% (6/41) studies with dropout rates 10% or lower, and 3 (7%, 3/41) with dropout rates greater than 50% [30,35,36,67]. A regression analysis revealed that there is no strong correlation (R^2^=.0639) between the study period and dropout rates. The next section compares dropout rates with payments.

##### Payments and Dropout Rates

**Figure 5:**
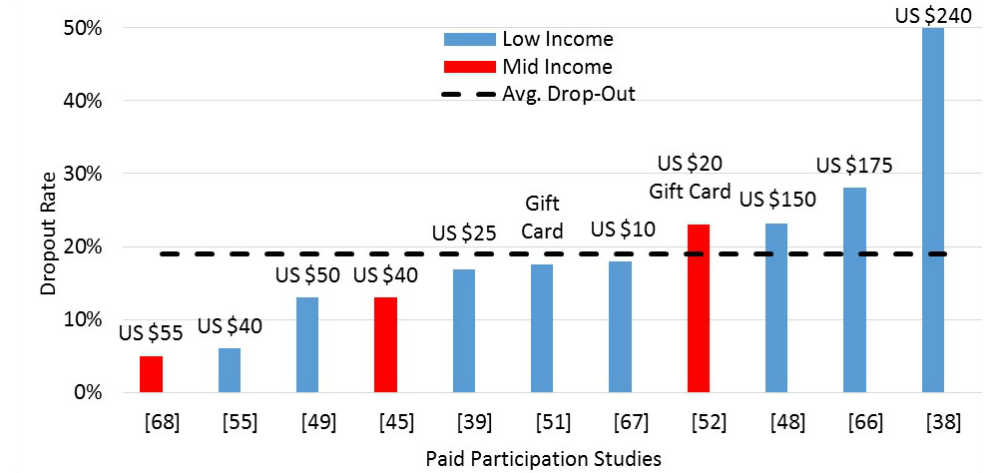
Dropout rates for paid participation studies.

There were 26% (11/41) studies that paid individuals for participation and reported dropout rates (Figure 5). Eight of these were low-income populations and 4 exceeded the average dropout rate of 19%. Interestingly, the 3 highest paying studies [40,48,68] were all low-income and were above the 19% average dropout rate. Anderson et al [41] (low-income) compensated participants with US $25 (17% dropout) and Wakefield et al [53] gave a US $20 gift card for their cooperation at the end of the study (23% dropout). Katz et al [40] (low-income) relied heavily on texting glucose readings to physicians and gave active participants US $20 monthly cellphone waiver for 1 year if they entered glucose readings that month (50% dropout). Arora et al [68] (low-income) paid participants US $175 for participating 6 continuous months (28% dropout) and assisted 3 people by awarding US $20 monthly stipends to alleviate costs of upgrading mobile phone service to unlimited texting capability.

Arora et al [49] (low-income) paid US $50 after successful completion of a 3-week study (13% dropout). Heisler et al [56] (low-income) gave participants US $20 stipend per assessment, which consisted of taking HbA1C readings (6% dropout). The study by Grilo et al [69], which lasted a period of 6 months, paid US $10 for completing 3 months in the program (18% dropout). Davis et al [51] (low-income) gave a gift card for each completed clinical visit, but did not specify the gift card’s monetary amount (18% dropout). Dick et al [70] (mid-income) gave their participants US $25 to cover the expenses of unlimited text messaging plan and US $30 for their participation (5% dropout). Aikens et al [61] (low-income) gave US $20 for patients at baseline then another US $20 at study completion of the 3-6 month study (13% dropout). Finally, Ratanawongsa et al [48] (low-income) awarded individuals a US $25 gift card per participation and US $50 for each interview spaced 6 months apart over a 1-year period (23% dropout) up to US $150.

Payments amounts do not appear to have a direct correlation with dropout rates, but this observation may be confounded by the fact that payment amounts are intended for different purposes in each study (eg travel, technology) and are a reflection of the effort required. For example, the highest paying study required a 2-year commitment for regularly entering glucometer readings.

**Figure 6:**
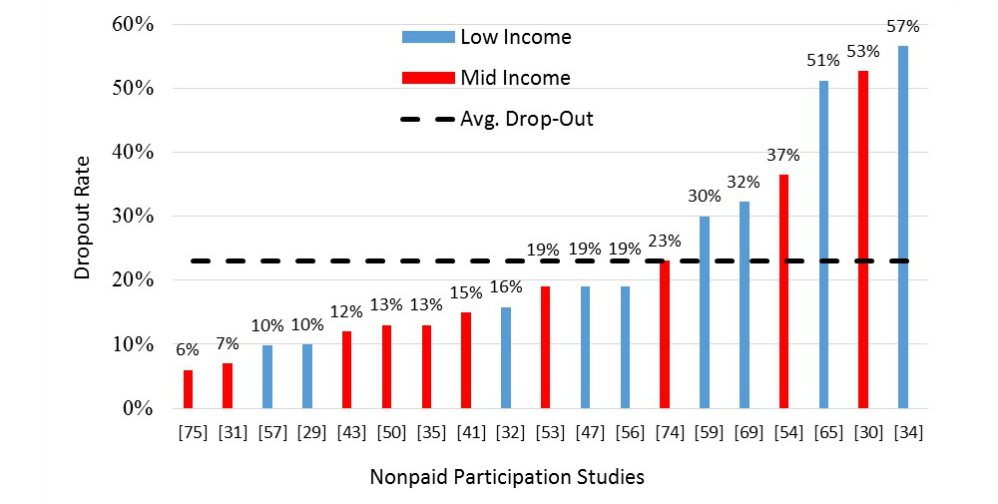
Dropout rates for nonpaid participation studies.

There were 46% (19/41) studies that did not pay for participation that also reported the dropout rate (Figure 6). The average dropout rate for nonpaid participation studies was 23% compared with 19% for paid participation studies.

##### Barriers and Dropout Rates

There was no strong relationship between high dropout rates and the number of barriers identified in each study. We suspect this lack of correlation is due to the journal articles not explicitly reporting on barriers and the low sample size of papers. However, we did note that studies with higher than average dropout rates had an average of 4.6 barriers, compared with those studies with lower than average dropout rates having an average of only 3.5 barriers.

##### Suggestions to Reduce or Eliminate Barriers

Declining engagement or participation (a symptom of barriers) was the most commonly cited problem. Some studies reported that the intervention was time-intensive [40] and others simply attributed it to poor follow-up [33]. However, no data were recorded on why patients did not continue. Tang et al [37] and Heisler et al [56] proposed that personalized strategies to achieve health goals (eg, taking medication) might keep patients engaged. Studies with minority populations [71] suggested providing culturally tailored educational activities into the patient’s daily life, so that interventions occur at multiple levels and are conveniently delivered. A few studies suggested that declining participation could be attributed to the cost of maintaining the technology (eg, cell phone bills). One study involving mobile phone messaging [68] helped participants pay for unlimited text messaging, thereby eliminating the patient’s participation cost. But the dropout rate remained high at 28%. Carter et al [55] equipped patients with laptops containing wireless broadband cards for this same reason, but again dropout rates were high at 37%. To improve adherence to the new technology, more training and involvement is needed while communicating with patients [29,40].

#### Conceptual Model

**Figure 7:**
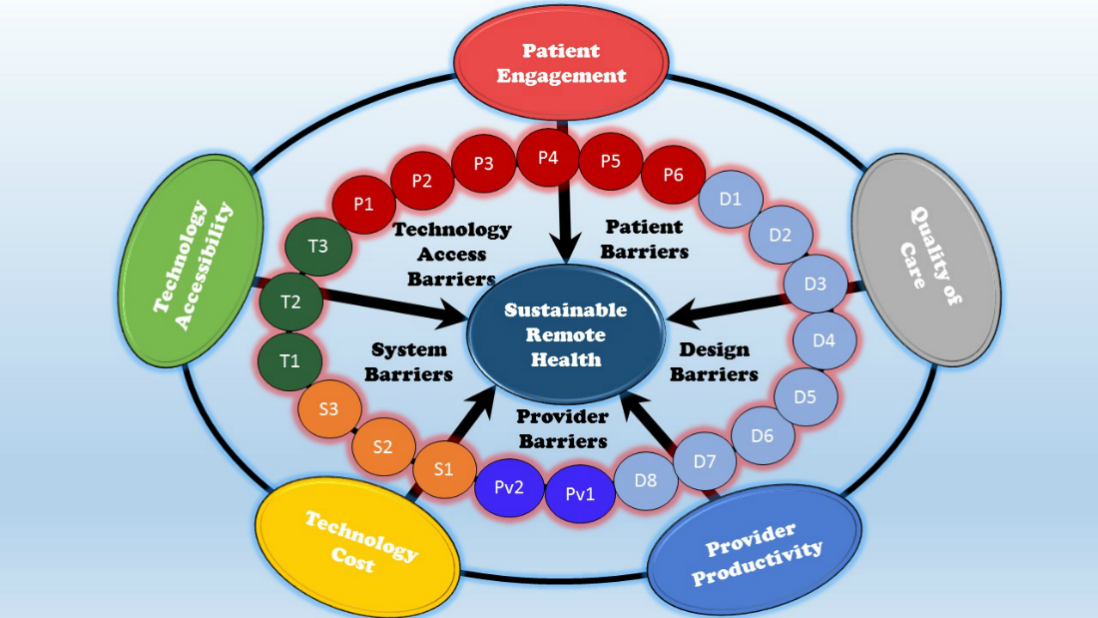
Conceptual model of barriers to successful, sustainable remote health.

The triple aim in health care is often referred to as *access, quality, and cost* [72]. There is increasing recognition that *patient-engagement* is critical to achieving high-quality, affordable care [73,74] due to the prevalence of chronic diseases that are behavior- and lifestyle-driven. Moreover, systems theory has shown that system *productivity* is a requirement to obtain quality control at an affordable cost [75]. *Provider productivity* is especially important in an era of aging population, growing chronic disease, and decreasing primary care resources. We used these 5 elements to develop a conceptual model (Figure 7) for effective remote health, adapting it around technology and organizing it around the 2 main players in the system, patients, and provider. We included insurance companies in the provider side for parsimony in the model. We then mapped the barriers into these elements. The mapping is not one-to-one, as some barriers affect multiple elements. We believe these elements are 5 necessary conditions for successful and sustainable remote health. Although we do not argue that the list is complete or that meeting these conditions is sufficient for sustainable success, we do believe that many of the challenges of remote health would begin to dissolve if these conditions were achieved. We now discuss how the barriers identified in this review affect these 5 elements.

##### Patient Engagement

The remote health intervention of the future must ensure regular *patient engagement* to promote positive behavior, efficacy, and retention. Designing a remote health intervention with strong patient engagement will favorably affect patient dropout rates. Unfortunately, this category had the largest number of barrier instances with 60 identified (Barriers T3, P1-P6, D1, D3, D5, and D6) in the systematic review. Barriers to patient engagement include low formal education, technology illiteracy, and health illiteracy on the part of the patient, as well as low perceived value of the system, a preference for personal contact with their provider, and medication nonadherence. Also, lack of Internet accessibility in the patient’s area, lack of customization of the system design, poor content choices, inconvenient frequencies of interventions, and lack of inclusion in the system design are also barriers to high levels of patient engagement. Clearly, if patients cannot or will not engage in remote care, remote health systems will fail.

##### Quality of Care

It is important not to compromise on quality of care delivered by the remote health intervention. The system must provide high *quality of care* that leads to improved health outcomes, improved perceptions of care, and higher efficacy. There were 28 instances (Barriers D2, D4, D5, D7, and Pv1) of barriers that threaten the ability to provide patients with quality care, which include lack of accuracy of data (eg, manual input), failure to understand the impact of remote health interventions on comorbid conditions, timing, and frequency of intervention and patient-provider–interactions, and accessibility of patient data records to the provider. Again, if quality of care cannot be assured, remote health will not succeed.

##### Provider Productivity

The remote health intervention must improve *provider productivity* so that a diminishing number of providers can care for an increasing number of patients. There were 23 instances (Barriers D4, D8, Pv2, and S1) of provider productivity barriers identified in the systematic review. Remote health interventions that are labor-intensive, require a high number of patient-provider–interactions, have poor integration into the provider work flow, and are limited in scalability contain barriers to increased provider productivity and cannot be sustained.

##### Technology Accessibility

Remote health interventions must be *accessible* for the patient. Accessibility implies technology that is affordable to the patient and ubiquitous at the point of use to enhance adoption and retention. The systematic review identified 16 instances (Barriers T1-T3) in which technology accessibility to the patient was a barrier because the patient did not already have the technology, could not afford the technology, or had limited Internet access. Technology accessibility must be achieved for remote health to have an impact.

##### Technology Cost

The remote health intervention will need to be *cost-effective* for long-term sustainability. This implies an overall reduction in health care spending, due to reduced emergency department and hospitalization, which exceeds the capital costs providers must pay to implement and maintain the remote system. The systematic review identified 16 instances (Barriers S1-S3) of barriers to cost-effective technology, which include limitations on scalability, lack of program reimbursement by insurance, and high-cost of interventions. Remote health systems that do not pay for themselves will not be sustainable.

### Risk of Bias Within and Across Studies

All the articles included in the systematic review had their own internal biases. ** **Mainly, remote health approaches rely heavily on the data submitted by the patients [67,70,76]. These data can be willingly distorted or abstained by patients. Other patients may have the best self-reporting intentions, but could have been confused on the proper way to use the remote health intervention [34,59]. In addition, the outcomes were not always reliable. For example, Bell et al [77] counted the number of diabetes videos watched by patients each month. However, there is a big difference between “watching” a video and actually “paying attention” and “understanding” a video. Other studies had selection bias, especially all the VA studies because the conclusions for this population group are not necessarily generalizable to the rest of the US population. Many were written with an optimistic perspective on remote health and did not take a critical viewpoint in addressing the challenges encountered during implementation. Even if the barriers were noted during a study, the authors of the study may not have reported them in the journal article as they were not the focus of the publication or due to space limitations of the journal. Furthermore, the barriers identified in certain populations, such as patient barriers in mid-income populations, could be attributed to reporting bias by the researchers who assume patients are having difficulty adopting to technology instead of examining possible system or provider issues. Finally, many of the selected articles were pilot studies with 47% (20/41) having population sizes less than 100. In addition, 54% (22/41) of the studies were shorter than 6 months. Thus, the barriers identified are not those that would necessarily arise during large-scale, long-term implementation of a remote health intervention for type 2 diabetes self-management.

## Discussion

### Limitations

The authors recognize several limitations in this systematic review. Although several databases were searched, including databases with conference proceedings, as well as references of included studies, it is possible some articles fitting the inclusion criteria were missed. There are several descriptive terms for remote health and the authors do not claim that the search criteria included a comprehensive list of these terms. Identifying barriers was especially challenging as they were not always explicitly labeled and the authors had to use their best judgment to identify, interpret, and classify the barriers in each study. When coding the articles, the authors may have overlooked some barriers because of the manner in which they were described in the article. In some instances, the authors had a difficult time agreeing whether some issues were in fact barriers, or a symptom of a barrier (eg, patient engagement); thus, the identification and determination of barriers were subjective and could have been overlooked by a particular reviewer. The final list of barriers collected is not necessarily comprehensive but was created based on findings from the included studies. Finally, for study characteristic results, some studies only had limited information available such as not specifying the type of blood pressure (systolic or diastolic) used or not specifying the device used for communication via email and Internet.

### Conclusions

This systematic review analyzed papers with remote health interventions for type 2 diabetes patients. Technology in remote health reduces the burden of diabetes by providing patients with medical resources and education without the need to leave their homes. Six databases were searched for peer-reviewed journal articles published between 2010 and 2015 that implemented a remote health intervention for type 2 diabetes care. A total of 53 papers were selected on 41 different studies. The principal findings of this systematic review included analysis on barrier classification and inventory. Lack of data accuracy was the most common barrier as it was identified in 32% (13/41) of all studies and was equally found in both low- and mid-income populations. The lack of data accuracy was often a result of manual reporting or input of monitoring data. Concerns over scalability were cited in 24% (10/41) of studies (mostly mid-income) and technology illiteracy was observed in 24% (10/24) of studies (mostly low-income). Declining patient engagement was observed in almost every study as a result of these barriers.

Few studies addressed mechanisms for reducing barriers. For those that did, suggestions were made to customize the strategies or provide culturally tailored solutions to increase patient engagement. Some studies thought that assistance with technology education or cost would reduce dropout rates. Around 29% (12/41) of the studies paid patients for participation. Some reviews maintain that there is insufficient evidence to conclude that remote health interventions significantly improved type 2 diabetes outcomes [13]. The total cost was only reported in a single study, thus leaving open questions on cost-effectiveness of remote health. A review by Radhakrishnan et al [26] was most similar to ours because it identified barriers and facilitators for sustainability of telehomecare programs for chronic disease management. Although some barriers were common to both (including health literacy and cost-effectiveness), our review identifies many additional barriers, including scalability, provider training, and system design.

We also proposed a conceptual model for successful implementation of remote health interventions. The model explains that technology accessibility, increased patient engagement, technology cost, increased provider productivity, and increased quality of care are 5 necessary conditions for remote health. Focusing on the barriers that impede these necessary conditions (eg, technology illiteracy and data accuracy) will better connect the patients to the clinics and providers for successful implementation of a remote health intervention for diabetes self-management in the United States. The results of this systematic review will facilitate other research in the design of remote health technology interventions as we identify common impediments in the design, implementation, adoption, and communication of remote health for diabetes patients. Specifically, technology must advance to improve reporting accuracy and reliability of the data communicated from the patient to the provider. Identifying ways to address the scalability of remote health interventions should also be a priority, as well as innovative designs that allow customized interventions and increase patient engagement. The barrier inventory provides visibility and evidence that these are the most dominant, pressing challenges facing the advancement of remote health today.

## Appendix

Multimedia Appendix 1 Search terms used for Medline and CINAHL.

Multimedia Appendix 2 Summary of 41 studies in systematic review showing the author names, income levels of patient participants, terminology used, study type, technology used, outcome measures, and barriers.

Multimedia Appendix 3 Statistics for study characteristics.

## Abbreviations

BMI: body mass index
CINAHL: Current Index to Nursing and Allied Health Literature
DBP: diastolic blood pressure
eHealth: electronic health
EHR: electronic health record
GHb: glycated hemoglobin
HbA1C: glycated hemoglobin
HDL: high-density lipoprotein
IDEATel: Informatics for Diabetes and Education Telemedicine
LDL: low-density lipoprotein
MAC: Medical Assistant Coaching
mHealth: mobile health
PHQ-9: Patient Health Questionnaire Depressive Screen
RCT: Randomized Control Trial
SBP: systolic Blood Pressure
SMS: short message service
TExT-MED: text message–based mobile health intervention
