# Supplementary material for: Barriers to Remote Health Interventions for Type 2 Diabetes: A Systematic Review and Proposed Classification Scheme
Source: J Med Internet Res. 2017 Feb 13;19(2):e28. doi: 10.2196/jmir.6382 (PMC5329647; doi:10.2196/jmir.6382)
Supplement: Multimedia Appendix 1 [file jmir_v19i2e28_app1.pdf]

Search terms used for Medline and CINAHL.

Medline Search
1. exp Telemedicine/ or exp Remote Consultation/
2. ((remote adj1 health) or mhealth or telehealth or ehealth).ti,ab.
3. (telemonitor* or telemedicine).ti,ab.
4. or/1-3
5. exp Diabetes Mellitus, Type 2/
6. (diabetes adj1 (type 2 or type ii)).ti,ab.
7. (non insulin$ depend$ or noninsulin$ depend$ or noninsulin?depend$ or non insulin?depend).tw,ot.
8. ((typ$ 2 or typ$ II) adj3 diabet$).tw,ot.
9. or/5-8
10. 4 and 9
11. limit 10 to yr="2010 -Current"

EMBASE Search
1. exp telemedicine/
2. ((remote adj1 health) or mhealth or telehealth or ehealth).ti,ab.
3. (telemonitor* or telemedicine).ti,ab.
4. or/1-3
5. exp non insulin dependent diabetes mellitus/
6. (diabetes adj1 (type 2 or type ii)).ti,ab.
7. (non insulin$ depend$ or noninsulin$ depend$ or noninsulin?depend$ or non insulin?depend).tw,ot.
8. ((typ$ 2 or typ$ II) adj3 diabet$).tw,ot.
9. or/5-8
10. 4 and 9
11. limit 10 to yr="2009 -Current"
12. limit 11 to english language

CINAHL Search

(MH "Telemedicine+") OR (MH "Telehealth+") OR AB (((remote n1 health) or mhealth or telehealth or ehealth)) OR AB ((telemonitor* or telemedicine)) AND ((MH "Diabetes Mellitus, Type 2") OR AB ((diabetes n1 (type 2 or type ii))) OR AB ((noninsulin depend* or non insulin*)) OR AB (((type 2 or type II) n3 diabet*))
