# Supplementary material for: Barriers to Remote Health Interventions for Type 2 Diabetes: A Systematic Review and Proposed Classification Scheme
Source: J Med Internet Res. 2017 Feb 13;19(2):e28. doi: 10.2196/jmir.6382 (PMC5329647; doi:10.2196/jmir.6382)
Supplement: Multimedia Appendix 2 [file jmir_v19i2e28_app2.pdf]

Summary of 42 studies in systematic review showing the author names, income levels of patient participants, terminology used, study type, technology used, outcome measures, and barriers.

Author(s)	Income	Terminology	Study Type	Technology Used	Outcome Measures	Barriers
Mayes et al [33]	Low	Telecom-munications	Longitudinal study	Phone-Voice, Mobile Device -Internet/Apps, Glucose Monitor, Video, Email (Computer or Phone), Computer/Internet	HbA1C, SBP, DBP, weight/waist circumference	None
Dang et al [28]	Low	telemedicine telehealth	Quasi-experimental study, pilot study	Phone-Voice, Email (Computer or Phone), Remote Health Unit, Other Health Device	HbA1C, LDL, HDL, SBP, DBP, diet, form of exercise, physical changes/exams, Framingham risk score	P2
Carter et al [55]	Low	telehealth	RCT	Video, Computer/Internet	BGL, BMI, HbA1C, SBP, DBP, diet, form of exercise, self-efficacy/adherence, diabetes management practices scale, self-perception change	P7<br>T2, T3, T4<br>S4
Anderson et al [41]	Low	telehealth	RCT	Phone-Voice, Glucose Monitor	BMI, HbA1C, LDL, HDL, SBP, DBP, diet, form of exercise, physical changes/exams, survey, triglycerides/ cholesterol, depressive symptoms	P4<br>D2, D9<br>Pv2
Katz et al [40]	Low	mHealth	Pilot study	Mobile Device -Internet/Apps, Glucose Monitor	HbA1C, SBP, DBP, foot checks, self-efficacy/adherence, physical changes/exams, triglycerides/ cholesterol, eye exams, immunizations	P2, P6<br>T2<br>D3, D8<br>Pv1, Pv2<br>Pv3<br>S4
Arora et al [49]	Low	mHealth	Pilot study	Phone-Voice, Mobile Device -Internet/Apps, Phone-Text	HbA1C, foot checks, diet, form of exercise, self-efficacy/adherence, health information sources survey, desired content of educational materials survey	T1<br>D1, D3<br>D7, D9
Osborn, Mulvaney [29]	Low	text messaging intervention, voice intervention	Pilot study	Phone-Voice, Phone-Text	HbA1C, self-efficacy/adherence, helpfulness rating survey, Diabetes Medication Knowledge Questionnaire, Medicines for Diabetes Questionnaire, Medication Adherence Self-Efficacy Scale	P3, P5<br>T1<br>D3, D5<br>D8
Lyles et al [47]	Low	automated telephone	Substudy of RCT	Phone-Voice	trigger safety concerns and type analysis, health literacy	P6

					survey, self-perception change	D2, D5<br>Pv3
Arora et al [68]	Low	mHealth	RCT	Phone-Text	HbA1C, SBP, DBP, weight/waist circumference, alcohol intake	T1<br>D2
Heisler et al [56]	Low	eHealth	RCT	Mobile Device Internet/Apps	HbA1C, SBP, DBP, self-efficacy/adherence, diabetes distress survey, medication decisional conflict, knowledge about anti-hyperglycemic medication, satisfaction with clarity of medication survey, satisfaction with helpfulness of medication survey	P1, P2, P6<br>D2<br>Pv3
Grilo et al [69]	Low	telemoni-toring<br>telehealth	Pilot	Phone-Voice, Mobile Device -Internet/Apps, Email (Computer or Phone), Remote Health Unit, Computer/Internet, Other Health Device	BMI, SBP, DBP, self-efficacy/adherence	P6<br>D1
Iannitto et al [57]	Low	telemedicine telehealth	Pilot	Phone-Voice, Glucose Monitor	BGL, HbA1C, physical changes/exams, patient satisfaction survey	P1<br>D4<br>Pv1
Henderson et al [42]	Low	web-based	RCT	Phone-Voice, Glucose Monitor, Computer/Internet	BMI, HbA1C, LDL, SBP, self-efficacy/adherence, patient satisfaction survey, smoking status	P2, P4<br>T2, T3<br>D1, D8
Ruggiero et al [71]	Low	MAC	RCT	Phone-Voice, Mobile Device -Internet/Apps	BGL, BMI, HbA1C, foot checks, diet, form of exercise, efficacy/ adherence, depressive symptoms, survey, self-perception change	P3<br>T4<br>D1, D2<br>D4, D5<br>D6
Abebe et al [30]	Low	mHealth	RCT	Phone-Voice, Phone-Text, Mobile Device -Internet/Apps, Computer/Internet, Other Health Device	HbA1C, patient satisfaction survey	P2, P4<br>T1, T2, T3<br>D1, D8<br>D9<br>Pv2<br>S1, S2
Palmas et al [30]	Low	telemedicine televisits	RCT	Mobile Device -Internet/Apps, Video, Computer/Internet, Remote Health Unit, Other Health Device	BGL, BMI, HbA1C, LDL, HDL, SBP, DBP, foot checks, diet, form of exercise, self-efficacy/adherence, physical changes/exams, weight/waist circumference, depressive symptoms, statin use, Charlson Comorbidity Index, Lubben Social	P1, P2, P3<br>T4<br>D2, D6<br>D7<br>S3

					Network Scale, use of the home telemedicine unit, monitoring, diabetes health maintenance	
Davis et al [51]	Low	telehealth	RCT	Phone-Voice, Phone-Text, Video, Fax, Telehealth-Enabled Retinal Camera	BMI, HbA1C, LDL, SBP, DBP, physical changes/exams, weight/waist circumference, GHb, albumin-to-creatinine ratio	P1<br>S4
Ratanawongsa et al [48]	Low	automated telephone self-management support	Quasi-experimental study	Phone-Voice, Mobile Device -Internet/Apps, Email (Computer or Phone), Fax	HbA1C, LDL, SBP, DBP	P5<br>D2
Ruggiero et al [58]	Low	virtual world intervention	RCT	Computer Internet, Other Health Device	BGL, BMI, HbA1C, foot checks, diet, form of exercise, self-efficacy/adherence, summary of diabetes self-care activities, environmental barriers to adherence scale, fat-related diet habits questionnaire	P2<br>T1<br>D6, D9
Stone et al [43]	Mid	telemonitoring	RCT	Phone-Voice, Remote Health Unit	HbA1C, LDL, HDL, SBP, DBP, triglycerides/cholesterol, weight/waist circumference	S1
Wakefield et al [54, 63]	Mid	telemonitoring telehealth telemedicine home health monitoring	RCT	Phone-Voice, Remote Health Unit	BGL, BMI, HbA1C, SBP, foot checks, diet, form of exercise, self-efficacy/adherence	P5<br>T4<br>Pv3<br>S1
McFarland et al [60]	Mid	telehealth	Non-randomized parallel control group study	Phone-Voice, Remote Health Unit	HbA1C	S1
Tang et al [35]	Mid		RCT	Mobile Device -Internet/Apps, Glucose Monitor, Email (Computer or Phone), Computer Internet, Video	HbA1C, LDL, SBP, DBP, weight/waist circumference, depressive symptoms, Framingham risk score	D3
Leichter et al [58]	Mid	telemedicine	RCT	Phone-Voice, Email (Computer or Phone), Computer Internet	BMI, HbA1C, LDL, HDL, SBP, DBP, triglycerides cholesterol, weight/waist circumference	None
Buis et al [34, 76]	Mid	mHealth	Pilot study	Phone-Text, Mobile Device -Internet/Apps, email (Computer or	BMI, weight/waist circumference, weekly activities	T2<br>D2, D3, D5<br>Pv3

				Phone), Computer Internet		
Aikens et al [50,59]	Mid	mHealth	Observational study	Phone-Voice, Glucose Monitor, Email (Computer or Phone), Other Health Device	BGL, SBP, DBP, foot checks, self-efficacy/adherence, diabetes distress survey, self-perception changes, depressive symptoms, physical component scale, mental component scale	P3, P6<br>D2, D3<br>S1
Stamp et al [65]	Mid	telehealth	Quasi-experimental study	Phone-Voice, Glucose Monitor, Remote Health Unit, Other Health Device	BGL, HbA1C, SBP, DBP	T1<br>S2, S4
Siminerio et al [32, 33]	Mid	telemedicine	RCT	Video, Computer/Internet, Other Health Device	HbA1C	D9
Bartlett et al [34]	Mid	telehealth	Pilot study	Email (Computer or Phone), Computer Internet	BMI, SBP, form of exercise, weight/waist circumference	D1, D8<br>D9
Katalenich et al [59]	Mid		RCT	Phone-Voice, Phone-Text, Mobile Device-Internet/Apps, Glucose Monitor, Computer Internet	HbA1C, self-efficacy/adherence, DQoL-Impact Score, DQoL-Worry: Social/ Vocational, DQoL-Worry: Diabetes Related, DQoL-Total Score	P3<br>T4<br>D1, D2<br>D3
Quinn et al [76]	Mid	mobile phone personalized behavioral intervention	Cluster-randomized clinical trial	Phone-Voice, Mobile Device -Internet/Apps, Glucose Monitor, Email (Computer or Phone), Remote Health Unit, Computer Internet, Other Health Device	BGL, HbA1C, LDL, HDL, SBP, DBP, triglycerides, cholesterol, diabetes distress survey, diabetes symptom inventory, depressive symptoms	T1<br>Pv1<br>S1
Bell et al [77]	Mid	mHealth, telemedicine	Prospective randomized trial	Mobile Device -Internet/Apps, Glucose Monitor, Video	HbA1C, SBP, DBP, SMBG measurement data	D3
Dick et al [70]	Mid	mHealth, text-message based program	Pilot study	Phone-Text	foot checks, self-efficacy/adherence	P2<br>D2, D5<br>S1
Toledo et al [65]	Mid	telemedicine teleconsulta-tion	Pilot study	Video, Computer Internet	HbA1C, teleconsultation satisfaction survey	P7<br>D7<br>S1

Wakefield et al; Koopman et al [53, 63]	Mid	telemedicine	RCT	Phone-Voice, Remote Health Unit, Computer/Internet, Other Health Device	HbA1C, SBP, foot checks, diet, self-efficacy adherence, survey, program continuation rate	P2, P5<br>T1<br>D1, D2<br>D8, D9(2)[a]<br>Pv2<br>Pv3(2)[a]<br>S3
Pressman et al [45]	Mid	telemoni-toring, diabetes care telemoni-toring device trial	RCT	Phone-Voice, Remote Health Unit, Computer/Internet, Other Health Device	HbA1C, LDL, SBP, DBP, self-efficacy/adherence, fructosamine blood level	None
Shane-McWhorter et al [50]	Mid	telemoni-toring, remote monitoring	Nonrandom-ized prospective observational study	Phone-Voice, Glucose Monitor, Remote Health Unit, Computer Internet, Other Health Device	BMI, HbA1C, LDL, SBP, DBP, self-efficacy/adherence	D2, D9<br>S3
Stone et al [44]	Mid	telemedicine telemoni-toring	RCT	Phone-Voice, Remote Health Unit	HbA1C, SBP, DBP	S1
Fischer et a [38]	Mid	eHealth	RCT	Phone-Voice	LDL	S1
Greenwood et al [23]	Mid	telehealth	Quasi-experimental study	Phone-Voice, Mobile Device -Internet/Apps, Computer Internet, Other Health Device	HbA1C, LDL, foot checks, dilated eye exam, microalbumin	P2<br>D3, D4<br>D5<br>S2
McMahon et al [66]	Mid	telephone care	Prospective, longitudinal, randomized trial	Phone-Voice, Glucose Monitor, Email (Computer or Phone), Computer Internet, Other Health Device	BMI, HbA1C, LDL, HDL, SBP, DBP, diabetes distress survey, triglycerides/cholesterol, weight/waist circumference	S1

[a] Parenthesis are used to indicate the number of barriers occurring in the “Other” category
