# Supplementary material for: Barriers to Remote Health Interventions for Type 2 Diabetes: A Systematic Review and Proposed Classification Scheme
Source: J Med Internet Res. 2017 Feb 13;19(2):e28. doi: 10.2196/jmir.6382 (PMC5329647; doi:10.2196/jmir.6382)

Statistics for study characteristics.

Figure 8. Types of studies.

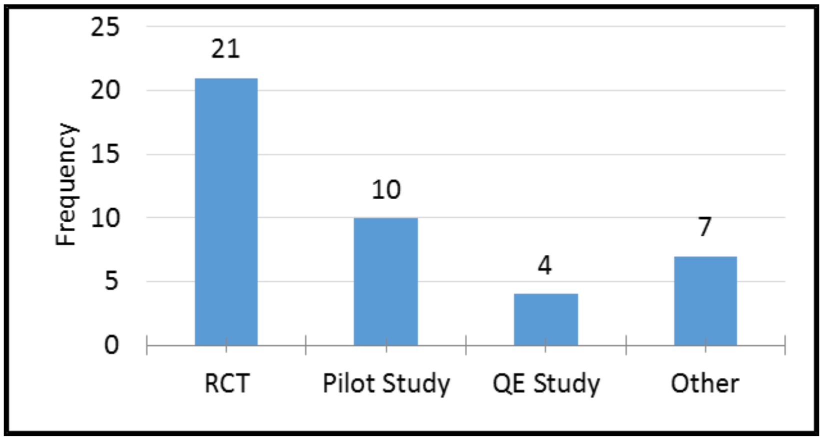

Figure 9. Length of study.

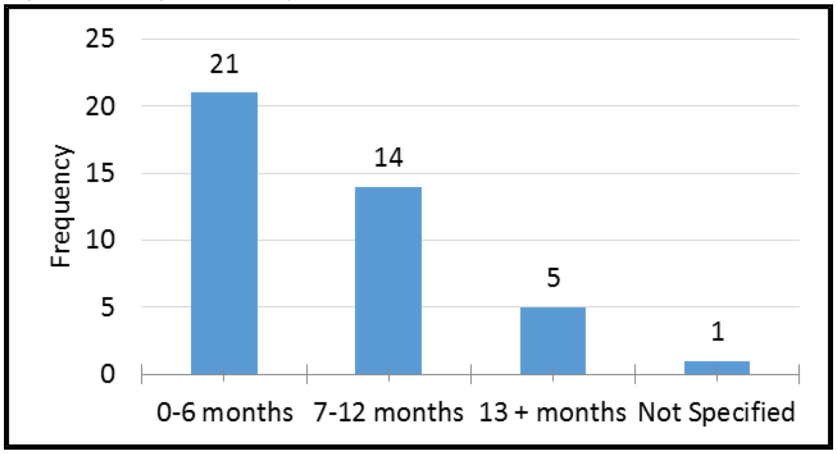

Figure 10. Number of participants.

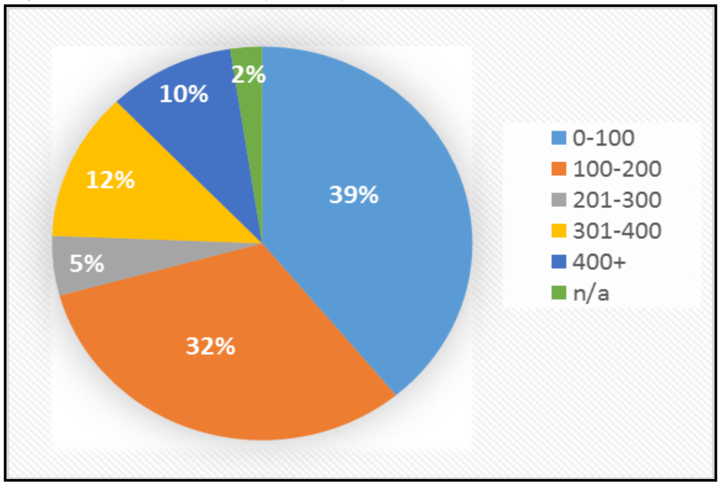

Figure 11. Mean participant age.

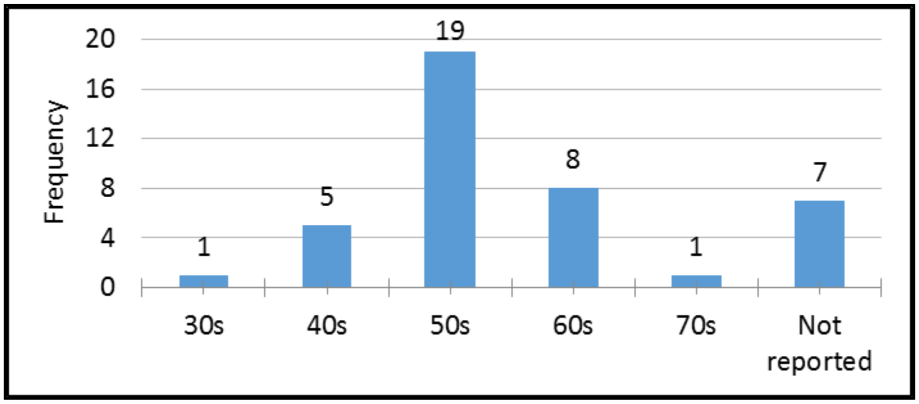

Figure 12. Studies by geographic region.

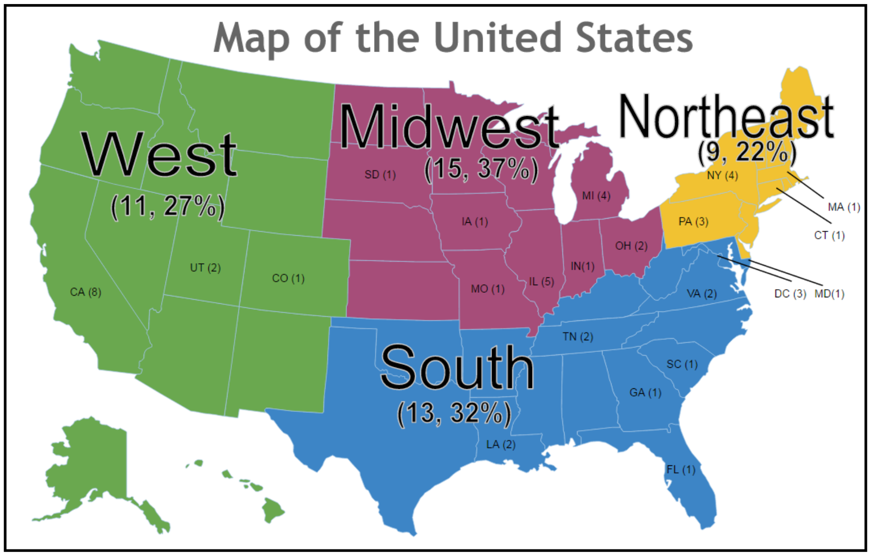

Figure 13. Comorbidities.

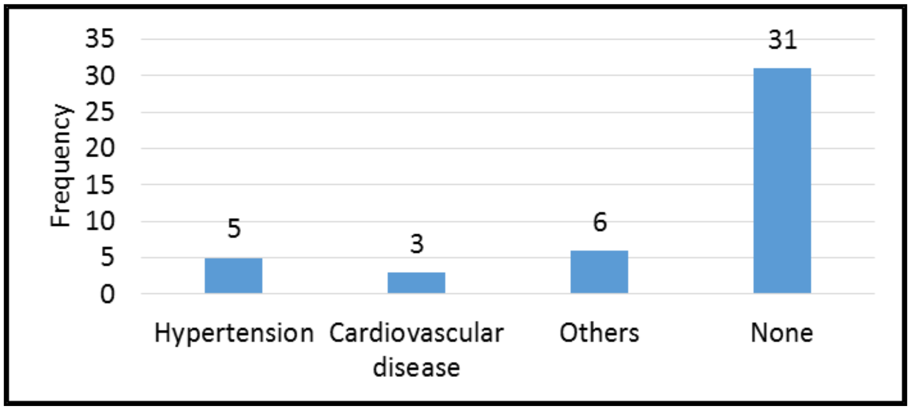

Figure 14. Histogram of dropout rates.

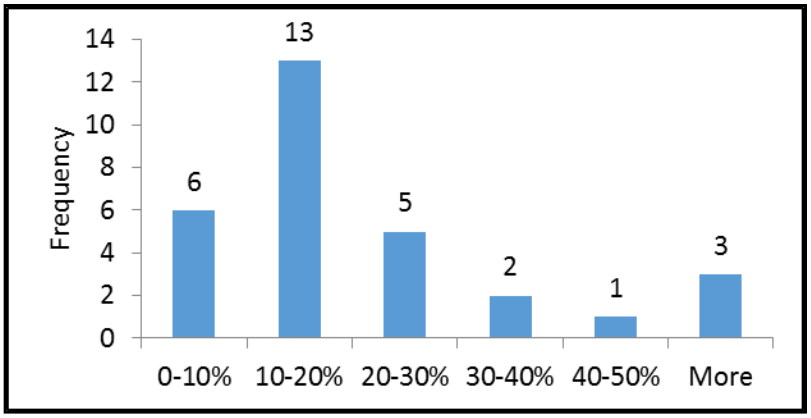

Supplement: Multimedia Appendix 3 [file jmir_v19i2e28_app3.pdf]
